# Comparing Motivational Interviewing with Education for Later Life Planning: A Two-arm Randomised Controlled Trial

**DOI:** 10.1093/geroni/igaf122.4296

**Published:** 2025-12-31

**Authors:** Youjuan Zhang, Xue Bai

**Affiliations:** The Polytechnic University of Hong Kong, Hong Kong, Hong Kong, China (People’s Republic); The Hong Kong Polytechnic University, Hong Kong, Hong Kong, China (People’s Republic)

## Abstract

The prevalent view of ageing as a period of decline often obscures opportunities for growth, agency, and continued contribution. Reframing ageing requires moving beyond simply providing knowledge and instead empower individuals to take proactive action in planning for a thriving later life. This study developed and evaluated a theory-informed intervention designed to reframe later-life planning as an empowering process across health, financial, social participation, family life, and long-term care domains through a randomised controlled trial. Community-dwelling adults (aged 50-75) in Hong Kong (63.0±3.9 years, 72.9% female, 74.6% retired) were randomised to a 4-session strength-based motivational interviewing (MI, n = 30) intervention resolving ambivalence, or a structured education intervention (n = 29) for knowledge/skill-building. Outcomes included attitudes, preparedness, stage of change and confidence in multidimensional later-life planning and psychosocial well-being, assessed at baseline (T0), immediately post-intervention (T1), and 1- (T2) and 3-month (T3) follow-ups. Generalised estimating equations examined differential changes in outcomes over time and groups. Overall, MI demonstrated sustained improvements in planning stages (p < 0.001, d = 0.77) and preparedness (p = 0.049, d = 0.33) at T3, outperforming Education. Such effects were particularly significant among social participation (planning stage: p = 0.02, d = 0.52) and health (preparedness: p = 0.03, d = 0.41) planning. Financial, family life, or long-term care planning, often requiring household-level consensus, were less responsive, warranting future family-based interventions. Both groups improved psychosocial outcomes (e.g., attitudes toward ageing, life satisfaction, self-efficacy; p < 0.05). This strengths-based model effectively empowers adults to engage with their later-life. By reframing planning from a burden to an opportunity for self-determination, it provides a tool for practitioners to advance equitable ageing.

